# Reviewed article: Micheletti VCD, Rosa RS, Dipp T, Silva LP, Campagnolo PDB, Mercaus LR, Brito JR, Bonin MEM. Cardiovascular risk and statin prescription in primary care: an observational study, São Leopoldo, 2018-2021. Epidemiol Serv Saude. 2025:34;e20240432

**DOI:** 10.1590/S2237-96222025v34e20240432.a

**Published:** 2025-10-20

**Authors:** Andréia Insabralde de Queiroz-Cardoso

**Affiliations:** 1 Universidade Federal de Mato Grosso do Sul, Campo Grande, MS, Brazil Universidade Federal de Mato Grosso do Sul Campo Grande MS Brazil

## Peer review A

## Questionnaire

Does the manuscript contain new and significant information to justify publication? Yes

Does the Abstract (Summary) clearly and accurately describe the content of the article? Yes

Is the problem significant and concisely stated? Yes

Are the methods described comprehensively? Yes

Are the interpretations and conclusions justified by the results? No

Is adequate reference made to other work in the field? No 

## Manuscript Structure

Length of article is: Adequate

Number of tables is: Adequate

Number of figures is: Adequate

Please state any conflict(s) of interest that you have in relation to the review of this paper. None

Interest: Good

Quality: Good

Originality: Good

Overall: Avarage

Recommendation: Major Revision

## Comments

Caros autores, seu manuscrito possuí informações importantes e que podem ser melhor elucidadas, faço algumas sugestões para melhorias.

De forma geral acredito que será necessário explicar melhor como os resultados podem ser generalizados e aplicados em outros contextos e justificar de forma mais clara a importância dos achados no contexto das doenças cardiovasculares no SUS e no Brasil.

Indico algumas alterações como:

1 - Melhorar a escrita do resumo, para deixar clara a conclusão com foco no objetivo apresentado.

2 - Introdução: Atualizar dados referente doenças cardiovasculares no Brasil.

3 - Método: retirar descrição do financiamento nos aspectos éticos.

4 - Discussão: pode ser melhorada com revisão da escrita e ampliar a discussão dos dados com novas evidências em prol da robustez e discussão embasada em melhores subsídios (artigo completo possuí apenas 17 referências). Retirar citação como “no estudo de Malta e colaboradores”. Rever também dados apresentados sem discussão em alguns parágrafos. Rever em todo o texto a utilização de primeira pessoa como: “sabemos”. Discutir melhor as prescrições de estatina e também as barreiras no acesso ao tratamento (Quais barreiras? Motivo? São os prescritores? O acesso ao medicamento?)

Deixar Claro qual limitação existe no estudo.

E no último parágrafo o qual deve ser curto e conciso, apresentar a conclusão da pesquisa com base do que se pode afirmar a partir dos resultados, com enfoque nos objetivos.

